# Sudden Cardiac Death in Systemic Sclerosis: Diagnostics to Assess Risk and Inform Management

**DOI:** 10.3390/diagnostics11101781

**Published:** 2021-09-28

**Authors:** Laura Ross, Elizabeth Paratz, Murray Baron, André La Gerche, Mandana Nikpour

**Affiliations:** 1 Department of Rheumatology, St. Vincent’s Hospital Melbourne, 41 Victoria Parade, Fitzroy, VIC 3065, Australia; m.nikpour@unimelb.edu.au; 2 Department of Medicine, The University of Melbourne at St. Vincent’s Hospital Melbourne, 41 Victoria Parade, Fitzroy, VIC 3065, Australia; elizabeth.paratz@svha.org.au (E.P.); andre.lagerche@baker.edu.au (A.L.G.); 3 Department of Cardiology, St. Vincent’s Hospital Melbourne, 41 Victoria Parade, Fitzroy, VIC 3065, Australia; 4 Clinical Research Domain, Baker Heart and Diabetes Institute, 99 Commercial Road, Melbourne, VIC 3004, Australia; 5 Department of Rheumatology, Jewish General Hospital, McGill University, 3755 Chemin de la Côte-Sainte-Catherine, Montreal, QC H3T 1E2, Canada; murray.baron.med@ssss.gouv.qc.ca

**Keywords:** systemic sclerosis (scleroderma), heart disease, cardiac, sudden cardiac death, arrhythmia

## Abstract

Cardiac disease is a leading cause of death in systemic sclerosis (SSc) and sudden cardiac death (SCD) is thought to occur more commonly in SSc than in the general population. Diffuse myocardial fibrosis, myocarditis and ischaemic heart disease are all prevalent in SSc and can be reasonably hypothesised to contribute to an increased risk of SCD. Despite this, SCD remains a relatively understudied area of SSc with little understood about SSc-specific risk factors and opportunities for primary prevention. In this review, we present an overview of the possible mechanisms of SCD in SSc and our current understanding of how each of these mechanisms may contribute to cardiac death. This review highlights the need for a future research agenda that addresses the underlying epidemiology of SCD in SSc and identifies opportunities for intervention to modify the disease course of heart disease in SSc.

## 1. Introduction

Systemic sclerosis (SSc) is a rare multi-system autoimmune disease with significant cardiac complications. The most common cardiac involvement is pulmonary arterial hypertension (PAH) and secondary right heart failure, which affects up to 15% of patients with SSc [1]. However, SSc has direct effects on the heart itself and primary SSc-associated heart involvement (SHI) is documented to affect all structures of the heart [2]. The aetiopathogenesis of SHI remains incompletely understood, with microvascular changes, inflammatory infiltrates and both interstitial and replacement fibrosis all observed in affected hearts [3,4]. Cardiac disease is a leading cause of death in SSc [5] but the nature of cardiac death remains poorly understood. Once clinically apparent with either heart failure or arrhythmia, SHI portends a very poor prognosis with a 60% survival rate three years from diagnosis [6]. In the largest SSc cohort study of over 5000 patients, SSc-myocardial disease accounted for 14% of overall deaths and non-SSc associated cardiovascular disease caused a further 12% of deaths [7]. The risk of death from SSc, and in particular heart disease in SSc, has remained largely unchanged for over 40 years [5]. This includes the risk of sudden cardiac death (SCD) which is reported to occur in up to 9% of individuals with SSc, [8,9,10,11,12,13,14,15] increasing to 67% in patients with clinically overt SHI [10].

**Figure 1:**
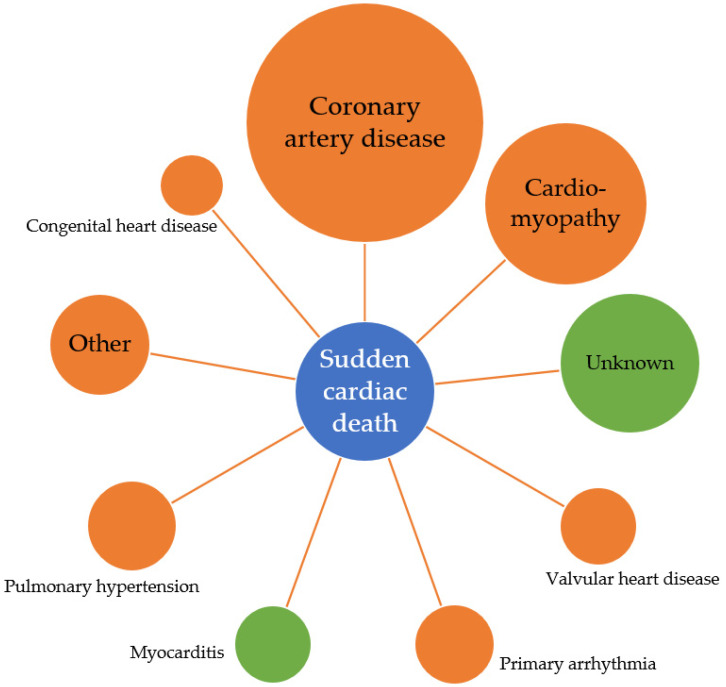
Causes of sudden cardiac death in the general population. Causes of sudden cardiac death highlighted in green reflect causes of sudden cardiac death seen most commonly in individuals aged <35 years.

The World Health Organisation defines SCD as a natural and unexpected death of cardiac cause within 1 h of the symptom onset or within 24 h of a person being last seen alive and well, in the case of an unwitnessed death [16]. Sudden cardiac arrest or SCD is the first presentation of cardiac disease in 50% of cases [17] and survival is low, with only 10% of patients with sudden cardiac arrest surviving to hospital discharge [18]. SCD accounts for almost half of all deaths from cardiovascular disease, with the largest proportion of SCD the result of ischaemic heart disease (IHD) [19,20]. Differences exist in the causes of SCD across the lifespan, with myocarditis and unexplained death more commonly observed in young populations, compared to coronary artery disease being the predominant cause in individuals over the age of 50 (see Figure 1) [21,22]. Importantly, individual risk prediction remains poor with most individuals who experience SCD not being identified as high risk prior to their arrest [20,23,24].

**Figure 2:**
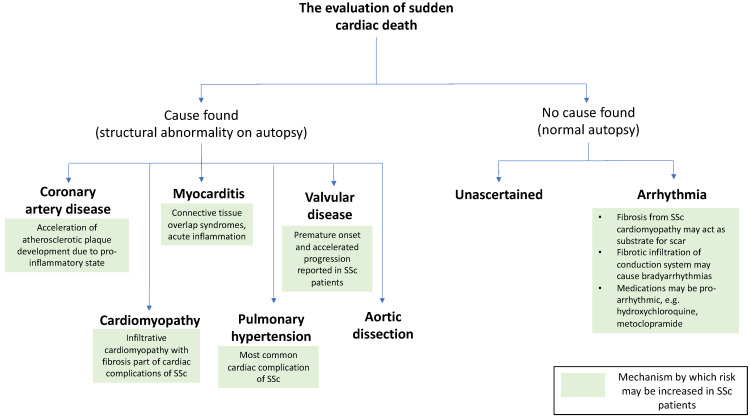
Potential mechanisms of sudden cardiac death in systemic sclerosis. *Abbreviations*: SSc: systemic sclerosis.

There have been no comprehensive population-based studies to define the epidemiology of SCD in SSc. The nature of SCD in SSc is not understood; it is unknown whether the direct effects of SSc on the myocardium or co-morbidities such as coronary artery disease account for sudden deaths of individuals with SSc. With consideration to what is understood of the underlying histopathological changes observed in SHI, Figure 2 summarises the potential mechanisms by which patients with SSc may be at increased risk of SCD.

In this article, we discuss the rationale and various mechanisms by which patients with SSc are at increased risk for SCD. In the absence of direct evidence for the effective treatment of SHI and disease-specific management of heart failure and arrhythmias in SSc, we propose a pragmatic approach to the risk assessment of patients with SSc and a research agenda to better understand the risk and primary prevention of SCD in SSc.

## 2. Coronary Artery Disease

Autoimmune inflammatory diseases are associated with an increased risk of coronary artery disease, hypothesised to be the result of chronic inflammation leading to accelerated atherosclerosis [25,26,27]. Unlike other autoimmune diseases, chronic inflammation is a less prominent feature of SSc, but the progression of the disease is characterised by an obliterative small vessel vasculopathy [28]. Important internal organ manifestations such as scleroderma renal crisis and PAH are a result of this small vessel vasculopathy. Microvascular manifestations of SSc have an unknown impact on the development of macrovascular disease and cardiovascular events. Endothelial dysfunction is common to the pathogenesis of SSc and atherosclerosis; therefore, the vasculopathy of SSc is hypothesised to accelerate atherosclerotic disease [29]. Early autopsy studies have demonstrated small coronary vessel vasculopathy with concentric intimal hypertrophy and cellular intimal proliferation in the absence of coronary artery atherosclerosis in patients with ante-mortem evidence of cardiac ischaemia [3,4,5,30,31]. In vivo, angina and clinical ischaemic heart disease in the absence of atherosclerotic coronary artery disease [32], and a subendocardial scar on cardiac magnetic resonance imaging (CMR) studies with normal coronary angiography have been observed [15,33,34].

Population-based studies have established that IHD is manifest more frequently in SSc compared to controls and IHD comorbidity with SSc is associated with poorer overall survival [29,35]. Whether this increased risk is due to microvascular dysfunction or accelerated atherosclerosis is unknown as no study has evaluated coronary angiography findings in SSc compared to non-SSc controls. A time-based risk assessment of IHD in SSc has indicated that patients are at the highest risk of acute myocardial infarction (AMI) within the first year of SSc diagnosis, with risk attenuating somewhat over time [36]. Microvasculopathy can be clinically prominent from early in the disease course and coupled with an inflammatory early phase of disease may explain this early excess risk of AMI. Non-invasive measures of atherosclerosis such as intimal media thickness and coronary calcium scores are elevated in asymptomatic SSc patients [37,38,39]. However, these scores were developed in non-SSc populations and have not been validated for SSc presenting difficulties in interpreting results according to current guidelines. Notably, autopsy studies have not found an increased rate of macrovascular coronary artery disease in SSc [3,4,5].

Given the increased frequency of IHD observed in the SSc population, it is reasonable to presume that a significant proportion of unexpected cardiac deaths may be due to IHD. Of high clinical importance, it is essential to understand whether atherosclerotic coronary artery disease is the underlying mechanism of death or whether the increased rates of IHD in SSc are driven by microvascular disease meaning that the SSc population has disease-specific causes of SCD.

## 3. Impaired Cardiac Function and Heart Failure

SCD is a frequent cause of death for patients with heart failure with reduced ejection fraction (HFrEF), however rates have reduced with optimization of medical therapy, cardiac resynchronisation therapy and use of implantable cardioverter-defibrillators (ICD) [40,41]. The cumulative risk of SCD in HFrEF is estimated to be 8.8% at 3-years in the modern treatment era [41]. Individual risk prediction tools for SCD in HFrEF remain limited, with an ongoing reliance on left ventricular ejection fraction (LVEF) to stratify patients into high and low risk categories [41]. Other risk factors for SCD in HFrEF are older age, male sex, lower systolic blood pressure, more severe symptoms, ischaemic cardiomyopathy and history of AMI, diabetes, elevated N-terminal pro B-type natriuretic peptide and renal dysfunction [41]. Notably, patients with HFrEF and SCD are less likely to be prescribed beta blocker therapy [41], which is a treatment that is often used cautiously in SSc due to concern about exacerbating Raynaud’s phenomenon and peripheral vascular complications of SSc.

HFrEF remains a feared manifestation of SHI with a cumulative survival of only 50% at 5-years [6]. Registry data suggest that reduced LVEF occurs in 5% of patients, [42] however this may underestimate the prevalence as registries have inherent survivor bias and 50% of patients who develop severe heart involvement do so within 3-years of SSc onset [6]. In addition to SHI itself, cardiac abnormalities including acute heart failure and severe pericardial disease can be triggered by scleroderma renal crisis [43]. SHI in combination with severe skin involvement has a particularly poor prognosis [44] and a high risk of SCD has been observed in patients with co-morbid skeletal myositis and heart failure [10]. The effects of cardiac function on survival in SSc are further re-enforced by the observation that more subtle measures of ventricular function such as global longitudinal strain are associated with mortality [45].

Diastolic dysfunction is observed more commonly in SSc than HFrEF and thought to be a result of myocardial fibrosis [46]. An increasing incidence of left ventricular diastolic dysfunction is observed throughout the SSc disease course and appears to independently predict increased mortality [47]. No direct relationship between diastolic dysfunction and risk of SCD in SSc has been reported.

Non-invasive diagnostic modalities such as CMR offer sensitive ways to detect myocardial fibrosis, the histopathological hallmark of SHI [3,4]. It is increasingly appreciated that there is a significant burden of myocardial fibrosis in SSc that is not manifest by either diastolic or systolic cardiac dysfunction [15,48,49]. CMR measures of both focal and diffuse myocardial fibrosis have been shown to predict the development of heart failure and arrhythmia, and a large burden of fibrosis is associated with increased mortality [14,15,50]. The prevalence of myocardial fibrosis is much greater than that of systolic dysfunction and reduced LVEF in SSc, despite the observed link between fibrosis and impaired cardiac function [50]. Specific cardiac predictors of those patients with myocardial fibrosis who will go on to develop HFrEF are unknown and as such there are insufficient data to recommend the routine use of CMR as a screening investigation for SHI.

## 4. Myocarditis

Overt myocarditis is infrequently reported in SSc, but when present may cause severe cardiac symptoms and is frequently associated with intractable heart failure and death [2]. However, silent myocarditis can be more frequently observed on CMR [51] and imaging studies of patients with cardiac symptoms, but no clinical myocarditis, show those with symptoms such as palpitations are more likely to have evidence of myocardial inflammation [52]. T2 mapping times, measuring diffuse myocardial inflammation, have consistently been shown to be elevated in SSc compared to healthy controls and are not associated with a measurable systemic inflammatory response or serum troponin levels [15,51]. A scoring system based upon CMR T2 ratio and percentage burden of late gadolinium enhancement has recently been proposed as a predictive tool to identify patients at heightened risk of future ventricular rhythm disturbance, [12] highlighting that even subclinical myocardial inflammation places SSc patients at increased risk of arrhythmia.

The risk of SCD appears to be significantly elevated in those with clinical evidence of myocarditis, with SCD or appropriate ICD discharge observed in up to 67% of patients [10,33,34]. As for the general population, when cardiac arrest occurs in patients with SSc-myocarditis, it is most commonly preceded by ventricular tachycardia or ventricular fibrillation [33,53]. Interestingly, fibrosis can be the most prominent histopathological finding on biopsy when myocarditis is suspected clinically and an increased risk of death has been observed with an increasing burden of fibrosis [10,33,53].

## 5. Arrhythmias

The exact physiological mechanisms linking SHI to arrhythmia are yet to be fully elucidated, with inconsistent reports of direct fibrotic infiltration of the conducting system itself as well as prominent areas of replacement and interstitial fibrosis throughout the myocardium thought to contribute a pathological substrate from which arrhythmias can arise [3,4,30,54,55]. Chronic ischaemia and inflammation likely also contribute to myocardial irritability in SSc. It is notable that observational data have shown that patients who receive vasodilator therapy with calcium channel antagonists appear to be at decreased risk of developing left ventricular systolic dysfunction and arrhythmia [13,42]. Whilst electrocardiogram (ECG) changes are associated with changes in cardiac function measurable by echocardiography, [56,57] left ventricular function is a poor predictor of the presence of arrhythmia as many patients with arrhythmias have preserved ventricular function [58].

Severe arrhythmic complications of SSc remain amongst the most feared disease manifestations but are fortunately a less common presentation of disease. Yet, despite their relative infrequency, arrhythmias account for a disproportionate number of SSc-associated deaths [7]. Non-specific arrhythmic and conduction abnormalities are detectable in 50% of SSc patients on resting ECG, and more frequently than other autoimmune conditions [56,58]. Yet, individual risk prediction for the progression to malignant arrhythmia remains limited and sudden cardiac arrest or death continues to be a first presentation of severe arrhythmic disease in SSc [59]. Both atrial and ventricular arrhythmias arise throughout the disease course, with patients remaining at risk of arrhythmic disease for the duration of SSc, with the exception perhaps of ventricular tachycardia which has been more frequently observed in young patients with shorter disease duration [56,60]. Consistently, a higher burden of ventricular ectopic beats has been associated with increased mortality [53]. Early publications of Holter monitor studies in SSc demonstrated a 6.2 fold increased risk of death if patients recorded > 1000 ventricular ectopic beats per 24 h period, [9] a finding that has remained unchanged over recent decades [8]. Prolonged QT interval is commonly reported in SSc, with a prevalence of 11–24.8% of patients [8,61]. QT prolongation is a hypothesised consequence of myocardial fibrosis and resultant disturbed ventricular repolarisation. Abnormalities of the QT interval can lead to a propensity to develop ventricular tachycardia and SCD [62]. In SSc, prolonged QT interval has been linked to features of more severe SSc [61,63,64] but whether QT interval can predict those patients who will go on to develop ventricular tachycardia or SCD is unknown.

Dysfunction of the autonomic nervous system can trigger ventricular arrhythmia and is associated with a five-fold increased risk of cardiovascular mortality and increased risk of SCD [65]. Autonomic dysfunction has been considered part of the spectrum of SHI [2]. A number of small studies have shown that impaired heart rate variability and turbulence, loss of circadian heart rhythm and impaired heart rate recovery after exercise can be commonly detected in SSc, from early in the disease course [66,67,68,69,70,71]. More recently, it was suggested that reduced heart rate variability in SSc is associated with impaired left ventricular strain [72]. Longitudinal data evaluating the prognostic importance of cardiac autonomic dysfunction is absent and given the unexpected nature of SCD, there is very little reporting of measures of autonomic dysfunction when such deaths occur. Reports of two sudden deaths following evaluation in one study found that these patients had markedly depressed circadian and heart rate variability and lower RR interval [70].

Rhythm abnormalities are treated empirically in SSc, with an absence of robust evidence-base for disease-specific treatments. Progressive cardiac disease is observed despite treatment with vasoactive, heart failure and anti-arrhythmic therapies. There are rare reports of successful treatment of sustained ventricular tachycardia with catheter ablation [73,74,75,76] and case series of successful ICD insertion with appropriate discharge and prevention of potential sudden cardiac arrest [77,78,79,80]. However, there are no SSc-specific guidelines for primary prevention of SCD in SSc with ICD.

## 6. Pulmonary Arterial Hypertension

Up to 15% of patients with SSc will be diagnosed with PAH over the course of their illness, with an increasing cumulative incidence associated with increasing disease duration [1]. Most deaths due to PAH are attributable to progressive right heart failure, with SCD the second commonest cause of death in PAH patients generally [81]. Little is known about the nature of SCD in PAH; ventricular arrhythmias are less commonly seen in patients with PAH compared to those with advanced left heart disease and other proposed mechanisms of SCD in PAH are compression of the left main coronary artery and pulmonary artery rupture or dissection [81,82,83]. Bradycardia and electromechanical dissociation were the two most commonly reported ECG findings in patients with PAH at the time of cardiopulmonary arrest, and are thought to portend poor prognosis in this patient group [83]. Supraventricular arrhythmias are increasingly recognised as a contributor to morbidity and mortality in PAH and commonly precipitate clinical decompensation. The incidence of supraventricular arrhythmias in PAH and the clinical association with important outcomes such as SCD remain relatively unexplored [81]. There is an absence of evidence to guide clinical practice in relation to screening for either atrial or ventricular arrhythmias in PAH, and no data to show any benefit from ICD insertion to prevent of SCD in PAH. The mainstay of PAH therapy is pulmonary vasodilator therapy, which has markedly reduced both mortality and requirement for heart-lung transplantation [84,85].

## 7. Valvular Heart Disease

It is hypothesised that there is an increased frequency of valvular heart disease due to the effects of SSc itself; however, it remains unclear whether the observed increased rates of aortic and mitral valve disease seen in population-based studies [86] are attributable to the increased frequency of screening echocardiograms performed as part of routine SSc care. Case-control studies have suggested an increased frequency of moderate to severe aortic and mitral valvular disease in SSc [87,88]. However, valvular heart disease has not been linked with increased mortality [87] and how valvular pathology may impact SCD risk in SSc is unstudied.

## 8. Predicting Risk of Sudden Cardiac Death in Systemic Sclerosis

There is no simple way to predict SCD in the general population, and therefore by extension in a population of SSc patients. The correct identification of all who are at risk of SCD has previously been described in European Society of Cardiology guidelines as ‘the philosopher’s stone of modern cardiology’ [89]. The nature of SCD is such that there will be some high-risk individuals who can be correctly identified as a result of clinical assessment and targeted imaging. Certain SSc disease features have been identified as placing individuals at increased risk of heart involvement and severe arrhythmia (see Table 1).

**Figure 3:**
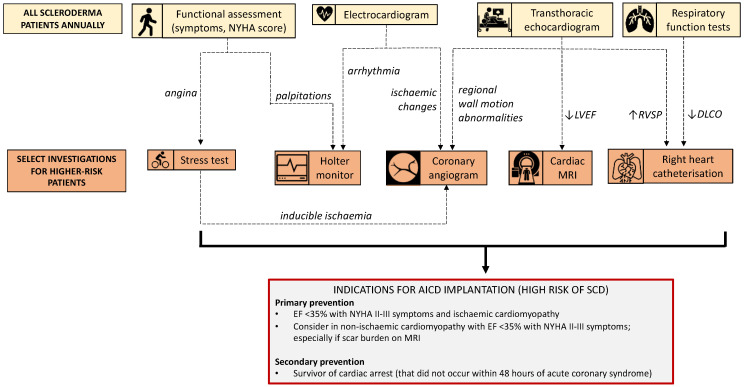
Cardiac evaluation of patients with systemic sclerosis. *Abbreviations*: DLCO: diffusing capacity of the lung for carbon monoxide; EF: ejection fraction; LV: left ventricle; MRI: magnetic resonance imaging; NYHA: New York Heart Association; SCD: sudden cardiac death; RVSP: right ventricular systolic pressure; SSc: systemic sclerosis.

Annual cardiopulmonary assessment is recommended as part of routine clinical care for patients with SSc [90]. Abnormalities detected by screening echocardiography and pulmonary function tests initiate further evaluation with second line investigations such as right heart catheterisation, CT chest and possible CMR (see Figure 3). Standardised asymptomatic testing that occurs as part of the routine management of SSc, as well as frequent contact with treating physicians, may create opportunities for the incidental identification of high-risk clinical features. Initial steps towards more sophisticated risk stratification of SSc patients have been made with the development of the Scleroderma Arrhythmia Clinical Utility Study (SAnCtUS) score, a four-category scoring system based on CMR findings to predict those patients at highest risk of ventricular rhythm disturbance [12]. In this particular study, patients with ≥4.6% burden of late gadolinium enhancement were found to have the greatest likelihood of having ventricular rhythm changes. These findings highlight the future role that more advanced imaging modalities such as CMR might play to inform our prognostication of SCD risk [91].

## 9. How Can Sudden Cardiac Death in SSc Be Prevented in the Future?

SHI is multifaceted, with vascular, fibrotic and inflammatory manifestations that can be reasonably theorised to contribute to an increased risk of SCD in SSc. However, the nature and exact risk of SCD in SSc remain undescribed. Knowledge gaps exist in sudden cardiac arrest and SCD research in general due to regulatory and study feasibility issues as well as the inherent nature of SCD itself [92]. To understand how SCD in SSc may be prevented in the future, more detailed studies are required both to quantify the burden of SCD and to describe the nature of SCD in SSc. Before risk factors are identified and targeted primary prevention strategies are developed, it needs to be understood if SCD in SSc is due to the disease itself or cardiac co-morbidities thought to more commonly co-exist with SSc. SHI is an uncommon manifestation of a rare disease; therefore, collaborative research efforts are crucial to gain insights and trial potential therapies and primary prevention strategies. These steps are essential to address the significant knowledge gaps in SSc and move towards preventing this catastrophic clinical event.

## Figures and Tables

**Table 1:** High risk features for systemic sclerosis heart involvement and severe heart involvement.

SSc Specific Risk Factors for Heart Involvement	High Risk Clinical Features for Severe Heart Involvement
Male sex	New unexplained breathlessness
Older age at SSc onset	Chest pain
Recently diagnosed SSc	Palpitations
Diffuse skin thickening, rapidly progressive skin thickening	Syncope or pre-syncope
Scl70 positive, U3RNP positive	Unexplained elevated troponin or BNP
Inflammatory myositis	LVEF <50%
Inflammatory arthritis Tendon friction rubs	Multifocal PVC or VE >1000 beats per 24 h

*Abbreviations:* BNP: B-type natriuretic peptide; LVEF: left ventricular ejection fraction; PVC: polymorphic ventricular contraction; Scl70: anti-topoisomerase I antibody; SSc: systemic sclerosis; VE: ventricular ectopic.

## Abbreviations

AMI	Acute myocardial infarction
CMR	Cardiac magnetic resonance imaging
CT	Computed tomography
ECG	Electrocardiogram
HFrEF	Heart failure with reduced ejection fraction
ICD	Implantable cardioverter-defibrillator
IHD	Ischaemic heart disease
LVEF	Left ventricular ejection fraction
PAH	Pulmonary arterial hypertension
SAnCtUS	Scleroderma Arrhythmia Clinical Utility Study
SCD	Sudden cardiac death
SHI	Systemic sclerosis heart involvement
SSc	Systemic sclerosis
